# p38 MAP Kinase Inhibitor Suppresses Transforming Growth Factor-β2–Induced Type 1 Collagen Production in Trabecular Meshwork Cells

**DOI:** 10.1371/journal.pone.0120774

**Published:** 2015-03-23

**Authors:** Miyuki Inoue-Mochita, Toshihiro Inoue, Tomokazu Fujimoto, Takanori Kameda, Nanako Awai-Kasaoka, Naoki Ohtsu, Kenichi Kimoto, Hidenobu Tanihara

**Affiliations:** 1 Department of Ophthalmology, Faculty of Life Sciences, Kumamoto University, Kumamoto, Japan; 2 Department of Ophthalmology and Visual Sciences, Kyoto University Graduate School of Medicine, Kyoto; 3 Department of Ophthalmology, Oita University, Yufu, Japan; Casey Eye Institute, UNITED STATES

## Abstract

Glaucoma is an age-related neurodegenerative disease of retinal ganglion cells, and appropriate turnover of the extracellular matrix in the trabecular meshwork is important in its pathology. Here, we report the effects of Rho-associated kinase (ROCK) and p38 MAP kinase on transforming growth factor (TGF)-β2–induced type I collagen production in human trabecular meshwork cells. TGF-β2 increased RhoA activity, actin polymerization, and myosin light chain 2 phosphorylation. These effects were significantly inhibited by Y-27632, but not SB203580. TGF-β2 also increased promoter activity, mRNA synthesis, and protein expression of *COL1A2*. These effects were significantly inhibited by SB203580, but not Y-27632. Additionally, Y-27632 did not significantly inhibit TGF-β2–induced promoter activation, or phosphorylation or nuclear translocation of Smad2/3, whereas SB203580 partially suppressed these processes. Collectively, TGF-β2–induced production of type 1 collagen is suppressed by p38 inhibition and accompanied by partial inactivation of Smad2/3, in human trabecular meshwork cells.

## Introduction

Glaucoma is an age-related neurodegenerative disease of retinal ganglion cells, and a leading cause of blindness worldwide. Elevated intraocular pressure (IOP) increases the risk of progression of visual dysfunction in glaucoma patients [1]. Increased resistance in the conventional aqueous humor outflow pathway is a major cause of elevated IOP in glaucoma patients [2]. In humans, the conventional outflow pathway is composed of the trabecular meshwork (TM), Schlemm’s canal (SC), collector channels, and episcleral veins. In this pathway, appropriate turnover of the extracellular matrix (ECM) in the TM is essential for maintaining normal outflow; excessive deposition of ECM increases the resistance to aqueous outflow [3], [4]. Various types of ECM exist in outflow tissues, including collagens, elastin, glycosaminoglycans, fibronectin and laminin. TGF-β2 increases the resistance in the aqueous humor outflow, via a mechanism involving deposition of ECM, including type 1 collagen, through changes in the actin cytoskeleton in the TM [5], [6], [7], [8], [9]. Furthermore, aqueous levels of TGF-β2 are increased in the eyes of glaucoma patients, compared to control patients [10], [11], [12]. Consequently, TGF-β2 is thought to be a key modulator of ECM turnover in the outflow tissues of glaucoma patients.

Canonical TGF-β2 signaling is mediated by Smad proteins. In this pathway, binding of TGF-β2 to its receptors phosphorylates, and thereby activates, receptor-regulated Smads (R-Smads), Smad2 and Smad3. Activated R-Smads form oligomeric complexes with a common-partner Smad protein, Smad4, before translocating into the nucleus, where they bind to the CAGA sequence and regulate target gene transcription. TGF-β2 signaling can also be transduced through collateral signaling pathways, such as the Akt-PI3K, p38 MAP kinase, ERK, and Rho-associated kinase (ROCK) pathways. Previous studies indicate that the ROCK pathway in TM cells is involved in controlling resistance in the aqueous humor outflow and thereby IOP [13], [14]. Mechanistically, ROCK inhibition increases aqueous outflow by dephosphorylation of myosin light chain (MLC)-2, leading to the disassembly of actin stress fibers and retraction of TM cells. Intriguingly, several recent clinical studies have demonstrated the IOP-lowering effect of ROCK inhibition in normal volunteers and glaucoma patients [15], [16], [17], [18], [19], and one of the drugs, Ripasudil was approved in Japan as an IOP-lowering drug. Thus, both ROCK and TGF-β signaling are considered important in the regulation of aqueous outflow and glaucoma pathology; however, it remains unclear how these signaling pathways relate to each other and relative effect of ROCK inhibitor to p38 MAP kinase inhibitor, a well-established inhibitor, on the ECM production in TM cells. Considering the suppressive effects of ROCK inhibitor on steroid-induced production of fibronectin and laminin in TM cells [20], the involvement of ROCK and other signaling molecules in the collateral pathways of TGF-β signaling and their potential as future pharmacological targets is of interest. Here, we report the effects of ROCK inhibitor and p38 MAP kinase inhibitor on TGF-β2–induced polymerization of the actin cytoskeleton and production of collagen type 1.

## Materials and Methods

### Materials

Recombinant human TGF-β2 was purchased from Wako Pure Chemical Industries (Osaka, Japan). Y-27632 and SB203580 were purchased from Merck Millipore (Darmstadt, Germany). The anti-COL1A2 (1:2,000 dilution;), anti-Caveolin 1 (1:200 dilution), and anti-myocilin (1:100 dilution) antibodies were obtained from Abcam (Cambridge, UK). The anti-MLC2 (1:800 dilution), anti-phospho-MLC2 (1:700 dilution; Thr18/Ser19), anti-Smad2/3 (1:1,000 dilution), anti-phospho-Smad2 (1:1,000 dilution; Ser465/467), anti-α-tubulin (1:1,000 dilution), and anti-lamin A/C (1:2,000 dilution) antibodies were purchased from Cell Signaling Technology (Danvers, MA). The anti-β-actin mouse monoclonal antibody (1:10,000 dilution;) was obtained from Sigma (St. Louis, MO). The anti-collagen 4 α 5 chain (1:50 dilution) and anti-matrix protein gla (1:400 dilution) antibodies were purchased from Santa Cruz (Dallas, TX). The tissue inhibitor of matrix metalloproteinase 3 antibody (1:1,000 dilution) was obtained from Thermo Pierce (Rockford, IL). The anti-vascular cell adhesion protein 1 antibody (1:100 dilution) was obtained from R&D systems (Minneapolis, MN).

### Cell culture

Primary human trabecular meshwork (HTM) cells were obtained from ScienCell (Carlsbad, CA), and maintained in Fibroblast Medium (FM; ScienCell) containing 5% fetal bovine serum and supplements (undisclosed growth factors and antibiotics; ScienCell), according to the manufacturer’s protocol. Cells cultured without serum were cultured in Dulbecco’s modified Eagle’s medium (DMEM; Wako) supplemented with 100 U/ml penicillin and 100 mg/ml streptomycin sulfate (Invitrogen, Carlsbad, CA) and GlutaMAX-I supplement (Life technologies). We used HTM cells between passages 6 and 8 in the present study. After 24 h of serum starvation, cells, with or without 30-min 10 μM Y-27632/SB203580 pretreatment, were treated with TGF-β2 without washout of Y-27632/SB203580.

### Rho activity assay

HTM cells were cultured on 10-cm dishes. After cells were grown to confluence, the culture medium was changed to serum-free DMEM, 24 h before TGF-β2 treatment. After treatment of the cells with 2.5, 5, or 10 ng/ml TGF-β2 for 30 min, RhoA activation was evaluated using a pull-down assay with a Rho Activation Assay Biochem Kit (#BK036, Cytoskeleton, Denver, CO), according to the manufacturer’s instructions. Densitometry of immunoblot membranes was performed using the Image J software (NIH).

### Immunocytochemistry

Glass coverslips in 12-well plates were coated with gelatin for 30 min at room temperature, and then washed with phosphate-buffered saline (PBS). HTM cells were grown on gelatin-coated glass coverslips, serum-starved for 24 h, pretreated with 10 μM Y-27632 or SB203580 for 30 min, and then stimulated with TGF-β2. After a 24-h period of stimulation, HTM cells were washed twice in PBS and then fixed in 4% paraformaldehyde in PBS for 15 min. After fixation, the cells were washed three times in PBS, permeabilized and blocked with 3% fetal bovine serum in PBS. Subsequently, the cells were incubated with primary antibodies overnight at 4°C, and then with Alexa-Fluor conjugated secondary antibodies (1:1,000 dilution; Life Technologies) for 1 h at room temperature. Phalloidin-FITC (1:400 dilution; Life Technologies) was used for F-actin staining. After cells were washed with PBS, they were mounted with VECTASHIELD mounting medium with 4',6-diamidino-2-phenylindole (Vector Laboratories, Burlingame, CA), and the slides were observed under a fluorescence microscope (Olympus BX51, Tokyo, Japan).

### Phagocytosis assay

HTM cells were grown in 12-well plates and incubated with pHrodo bioparticle red E coli (Invitrogen). Three hours later, Hoechst 33342 (Dojindo, Kumamoto, Japan) was added into the medium, and cells were investigated using a fluorescence microscope (Olympus).

### Western blot analysis

HTM cells were grown on 6-cm dishes, serum-starved for 24 h, pretreated with 10 μM Y-27632 or SB203580 for 30 min, and then stimulated with TGF-β2 for 24 hours or 100 nM dexamethasone for 72 hours (for the induction of myocilin). Then, the cells were washed three times on ice with ice-cold PBS, lysed with RIPA buffer (Thermo Scientific, Rockford, IL) containing protease (Thermo Scientific) and phosphatase inhibitors (Nacalai Tesque INC., Kyoto, Japan). Cell extracts were then centrifuged at 15,000 rpm for 10 min at 4°C. Supernatants were collected, and the protein content was determined using a BCA protein Assay Kit (Thermo Scientific). Samples were resolved using SDS-PAGE and subsequently transferred onto polyvinylidene difluoride membranes by electroblotting. After membranes were blocked with 2% ECL Advance Blocking Reagent (GE Healthcare, Little Chalfont, UK) in Tris-buffered saline containing 0.1% Tween-20 (TBS-T) for 30 min at room temperature, they were incubated with primary antibodies diluted with 5% Bovine Serum Albumin (WAKO) in TBS-T overnight at 4°C. After washing three times 3 times for 5 min each with TBS-T for 5 min, the membranes were incubated with horseradish peroxidase-conjugated anti-rabbit IgG (1:2,000 dilution; Cell Signaling Technology) or horseradish peroxidase-conjugated anti-mouse IgG (1:5,000 dilution; GE Healthcare), for 1 h at room temperature. After washing three times for 5 min each with TBS-T, signals were enhanced using a chemiluminescence system, ImmunoStar LD (Wako) and ECL Western blotting Detection Reagents (GE Healthcare), and exposed using a LAS-4000 EPUV Mini (FUJI FILM, Tokyo, Japan) imager. Densitometry of immunoreactive bands was performed using the Image J software (NIH).

### Nuclear and cytoplasmic extraction

Nuclear and cytoplasmic proteins were extracted from HTM cells using NE-PER Nuclear and Cytoplasmic Extraction Reagents (Thermo Scientific) according to the manufacturer’s protocol. Lamin A and α-tubulin were used to standardize nucleic and cytoplasmic protein level, respectively. Signals were detected by Western blotting as described above.

### Luciferase assay

Plasmid constructs used in these experiments were described previously [21], [22] HTM cells were seeded the day before transfection in 6- or 12-well plates. As an internal control, a plasmid containing Renilla luciferase (pRL-TK; Promega, Madison, WI) was co-transfected. After 24 h of transfection, the medium was changed to serum-free DMEM. 24 h later, cells were pretreated with 10 μM Y-27632 or SB203580 for 30 min, and then stimulated with TGF-β2 for a further 24 h. Transcriptional activity was assessed by transient transfection of a luciferase reporter gene fused with a 436-bp *COL1A2* promoter or 12 repeats of the Smad-binding element (CAGA 12). Transfection of HTM cells was performed at 80% confluence, using GeneJuice Transfection Reagent (Merck Millipore) according to the manufacturer’s protocol.

### RNA isolation and real-time polymerase chain reaction

Serum-starved HTM cells were stimulated with TGF-β2 for 24 h in the presence or absence of the 30-min pretreatment with 10 μM Y-27632/SB203580. Total RNA was extracted using NucleoSpin RNAII (Takara Biotechnology, Shiga, Japan), according to the manufacturer’s instructions, and was treated with DNAse I (Life Technologies) at 65°C for 10 min. The RNA was reverse transcribed using PrimeScript RT Master Mix (Takara Bio, Shiga, Japan), according to the manufacturer’s instructions. Quantitative real-time reverse-transcription polymerase chain reaction (PCR) was performed in 20 μl of reaction mixture containing 10 μl of PCR master mix (THUNDERBIRD SYBR qPCR Mix, TOYOBO, Osaka, Japan), 2 μl of cDNA samples and 0.3 μM primer pairs using Applied Biosystems 7000 (Life Technologies) according to the manufacturer’s instructions. The thermal cycling conditions were 95°C for 30 s, 40 cycles of 95°C for 5 s each, and 60°C for 31 s. All PCR reactions were performed in duplicate and β-glucuronidase (Takara Biotechnology; sequences were not disclosed) was used as a control. PCR was performed using the following primers: human COL1A2 (sense sequence, 5-GCA CAT GCC GTG ACT TGA GAC-3; antisense sequence, 5-CAT AGT GCA TCC TTG ATT AGG-3) and human fibronectin (sense sequence, 5-CAG GAT CAC TTA CGG AGA AAC AG-3; antisense sequence, 5-GCC AGT GAC AGC ATA CAC AGT G-3).

### Statistical analysis

Each experiment was repeated a minimum of five times. Data were analyzed by using the JMP version 8 statistical software package (SAS Institute, Cary, NC). All data represent the means of at least three independent experiments. Quantitative data were analyzed using the Tukey—Kramer HSD test. A P value < 0.05 was considered to indicate statistical significance.

## Results

### Activation of the Rho—ROCK signaling pathway in HTM cells by TGF-β2 stimulation

**Fig 1:**
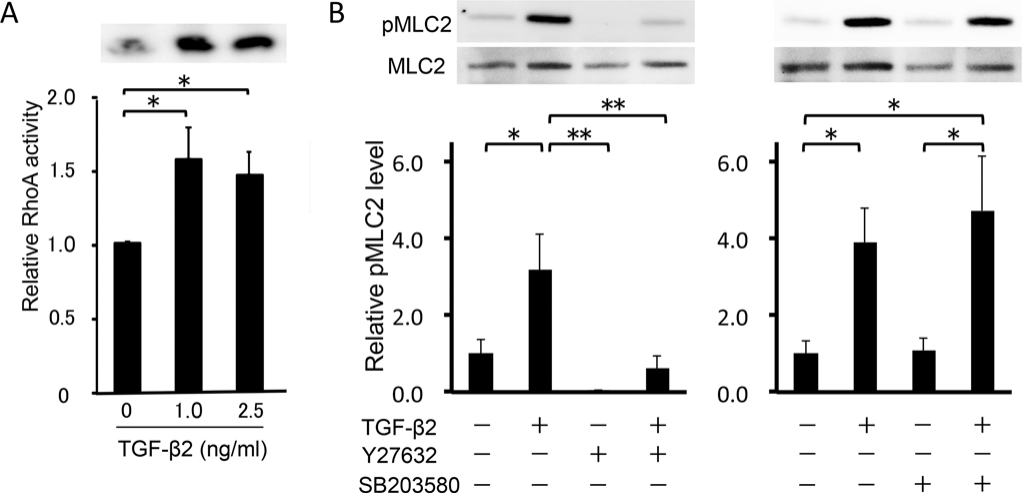
Analysis of the Rho signaling activity. (A) Effect of TGF-β2 on RhoA activity in HTM cells. (B) Effects of TGF-β2, Y-27632 and SB203580 on myosin light chain (MLC)-2 phosphorylation in HTM cells. Cells with or without 10 μM Y-27632 or 10 μM SB203580 pretreatment for 30 min were stimulated with 2.5 ng/ml TGF-β2 for 30 min. Data shown in upper panels are results of representative Western blot analyses of phosphorylated MLC2 (pMLC2) and total MLC2. Relative changes in the ratio of MLC phosphorylation are shown in the lower graph. Data are shown as means ± SE, n = 10. *P < 0.05 and **P < 0.01 calculated using the Tukey—Kramer HSD test.

**Fig 2:**
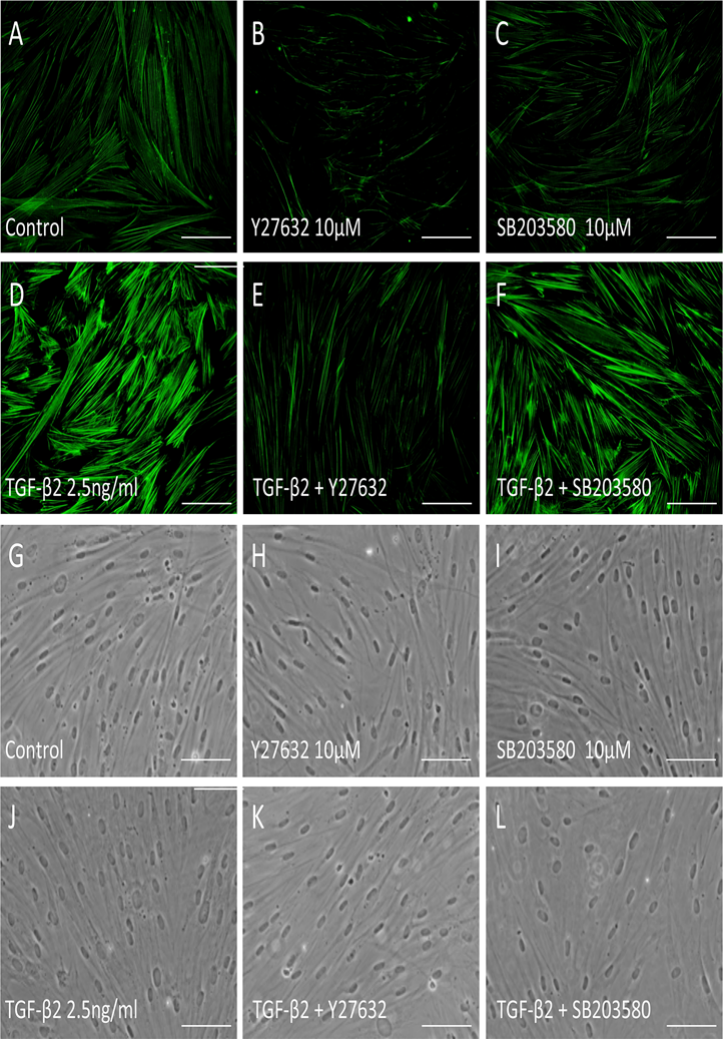
Effects of TGF-β2, Y-27632 and SB203580 on actin stress fibers in HTM cells. HTM cells were pretreated with 10 μM Y-27632 (B, E) or 10 μM SB203580 (C, F) for 30 min, and then stimulated with 2.5 ng/ml TGF-β2 (D-F) for 24 h, stained with phalloidin-FITC, and observed by fluorescence microscopy. Scale bar: 50 μm.

The characters of the cells in the present study were confirmed by expression of specific TM markers, phagocytosis function, and myocilin induction by dexamethasone (S1 Fig.). To elucidate the effect of TGF-β2 on the Rho—ROCK signaling pathway in HTM cells, we used a RhoA activity assay. Treatment with 2.5 ng/ml TGF-β2 for 15 min increased active RhoA 1.5-fold in HTM cells (Fig. 1A). The increase was not dose-dependent, and treatment with 5.0 ng/ml TGF-β2 did not produce a significant effect (data not shown). In the further experiments, we used 2.5 ng/ml TGF-β2 for stimulation of HTM cells to rigidly confirm the effects of the inhibitors. Next, we investigated the effect of TGF-β2 on the phosphorylation of MLC-2, a downstream effector of the Rho—ROCK signaling pathway. Stimulation with 2.5 ng/ml TGF-β2 for 24 h significantly increased the ratio of phosphorylated MLC-2 to total MLC-2 (95%CI: 0.169 to 4.192, P = 0.029; Fig. 1B). The effect of TGF-β2 on MLC-2 phosphorylation was inhibited to basal levels by 10 μM Y-27632, a specific ROCK inhibitor (95%CI: 0.551 to 4.573, P = 0.008; Fig. 1B), whereas the ratio was unaffected by 10 μM SB203580, a specific p38 inhibitor (Fig. 1B). Since phosphorylated MLC-2 induces polymerization of actin (F-actin), we examined the intracellular distribution of F-actin in HTM cells using fluorescent phalloidin. Increased formation of F-actin was observed after stimulation with TGF-β2. Furthermore, the effect of TGF-β2 on F-actin formation was inhibited by treatment with Y27632, but not SB203580 (Fig. 2A-2F). These results suggest that TGF-β2 induces actin polymerization through activation of the Rho—ROCK signaling pathway in HTM cells. The morphological change was not significant following the treatment with SB27632, while some cells presented retraction after treatment with Y27632 (Fig. 2G-2L).

### Effects on TGF-β2–induced type 1 collagen production in HTM cells

**Fig 3:**
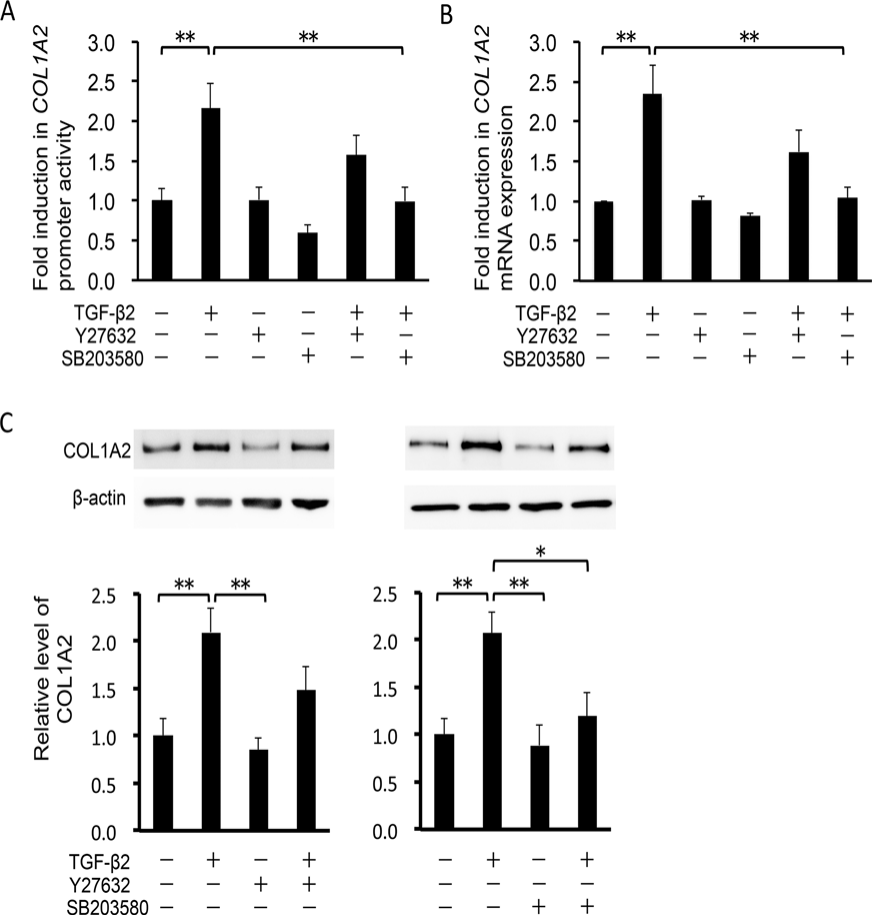
Effects of TGF-β2, Y-27632 and SB203580 on *COL1A2* induction in HTM cells. HTM cells were pretreated with 10 μM Y-27632 or 10 μM SB203580 for 30 min, and then stimulated with 2.5 ng/ml TGF-β2 for 24 h. Data are shown as means ± SE. *P < 0.05 and **P < 0.01 calculated using the Tukey—Kramer HSD test. (A) Effects of TGF-β2, Y-27632 and SB203580 on *COL1A2* promoter activity. Signals from a plasmid containing Renilla luciferase were used as an internal control. n = 15. (B, C) Effects of TGF-β2, Y-27632 and SB203580 on *COL1A2* and *Fibronectin* mRNA transcription, respectively. Relative expression levels of the genes were compared, using the comparative CT method. β-glucuronidase was used as a control. Relative signals from a luciferase reporter gene fused with 436 bp were compared. n = 6, each. (D) Effects of TGF-β2, Y-27632 and SB203580 on COL1A2 protein expression in HTM cells. Data shown in upper panels are results of representative Western blot analyses. Relative changes to β-actin are shown in the lower graph. n = 9.

To examine whether TGF-β2 enhances the transcriptional activity of *COL1A2*, encoding one of the chains of type 1 collagen, we assessed the activity of the *COL1A2* promoter using a luciferase assay. TGF-β2 significantly activated *COL1A2* transcription in HTM cells (95%CI: 4.273 to 41.869, P = 0.007; Fig. 3A). This effect was inhibited to near-basal levels by 10 μM SB203580 (95%CI: 4.385 to 41.981, P = 0.007; Fig. 3A) but not significantly inhibited by 10 μM Y-27632. Next, we investigated *COL1A2* mRNA expression by real-time RT-PCR analysis. Stimulation with 2.5 ng/ml TGF-β2 increased *COL1A2* mRNA levels 2.3-fold, compared with vehicle-treated control HTM cells (95%CI: 0.508 to 2.170, P < 0.001; Fig. 3B). Similar results were obtained in the Fibronectin mRNA expression (Fig. 3C). Pretreatment with 10 μM SB203580 significantly suppressed the TGF-β2-induced change in *COL1A2* mRNA levels (95%CI: 0.468 to 2.130, P < 0.001). In contrast, the effect of 10 μM Y27632 was not statistically significant on *COL1A2* mRNA levels in HTM cells. To confirm the effect of Y-27632 and SB203580 on type 1 collagen protein synthesis, we examined the expression level of COL1A2 using Western blot analysis. In TGF-β2–treated HTM cells, COL1A2 expression increased 2.1-fold compared with vehicle-treated control HTM cells (95%CI: 0.291 to 1.895, P = 0.004; Fig. 3D). TGF-β2–induced COL1A2 expression was significantly inhibited by pretreatment with 10 μM SB203580 (95%CI: 0.050 to 4.573, P = 1.701), whereas the effect of 10 μM Y-27632 was not significant. These data indicate that SB203580 inhibits TGF-β2-induced type 1 collagen production in HTM cells. In contrast, the effect of Y-27632 on this process is limited, suggesting that in HTM cells, Rho-ROCK signaling plays a lesser role in TGF-β2–induced COL1A2 expression than p38 MAP kinase signaling.

### Effects on the Smad signaling pathway

**Fig 4:**
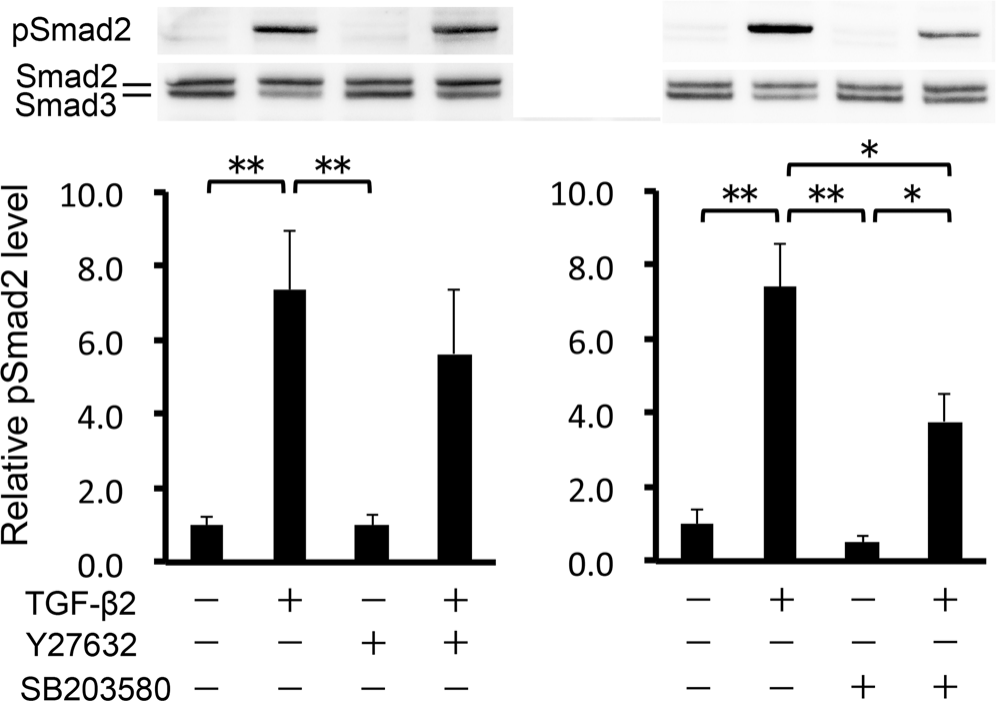
Effects of TGF-β2, Y-27632 and SB203580 on phosphorylation of Smad2 in HTM cells. HTM cells were pretreated with 10 μM Y-27632 (A) or 10 μM SB203580 (B) for 30 min, and then stimulated with 2.5 ng/ml TGF-β2 for 24 h. Data shown in upper panels are results of representative Western blot analyses of phosphorylated Smad2 (pSmad2) and total Smad2/3. Relative changes in the pSmad2 to total Smad2 expression level are shown in the lower graph. Data are shown as means ± SE, n = 6. **P < 0.01 compared with control using the Tukey—Kramer HSD test.

**Fig 5:**
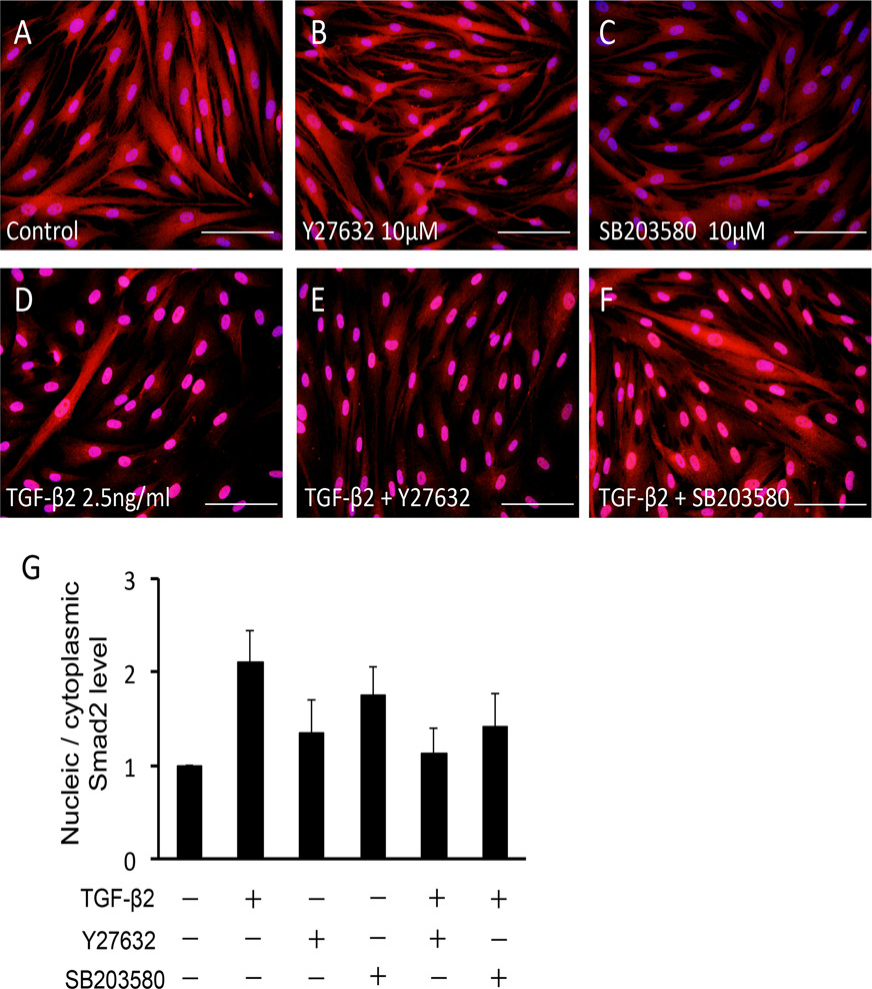
Effects of TGF-β2, Y-27632 and SB203580 on nuclear translocation of Smad2/3 in HTM cells. HTM cells were pretreated with 10 μM Y-27632 (B, E) or 10 μM SB203580 (C, F) for 30 min, and then stimulated with 2.5 ng/ml TGF-β2 (D-F) for 24 h, immunolabeled with anti-Smad2/3 antibody (red), and observed by fluorescence microscopy. Cell nuclei were counterstained by DAPI (blue). Scale bar: 50 μm. (G) Relative expressions of nucleic Smad2 to its cytoplasmic expression are shown in the graph. Nucleic and cytoplasmic proteins were extracted, and the expression level of Smad2 was assessed separately. Data are shown as means ± SE, n = 5.

**Fig 6:**
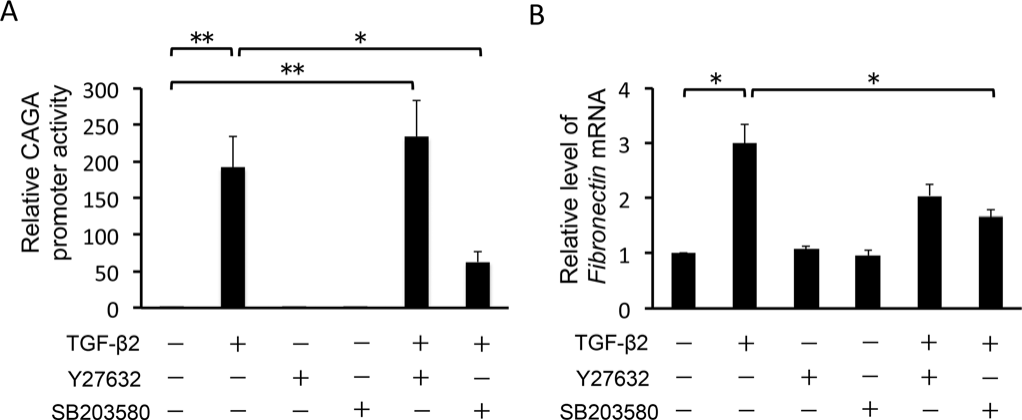
Effects of TGF-β2, Y-27632 and SB203580 on transactivation of Smad complexes in HTM cells. HTM cells were pretreated with 10 μM Y-27632 or 10 μM SB203580 for 30 min, and then stimulated with 2.5 ng/ml TGF-β2 for 24 h. Relative signals from a luciferase reporter gene fused with 12 repeats of the Smad-complex binding element (CAGA) were compared among samples. Signals from a plasmid containing Renilla luciferase were used as an internal control. Data are shown as means ± SE, n = 9. *P < 0.05 and **P < 0.01 calculated using the Tukey—Kramer HSD test.

Smads are the primary effectors downstream of TGF-β. TGF-β activates Smad2/3 by phosphorylation, thereby inducing their nuclear translocation and the resultant transcription of various target genes. Thus, we investigated whether 10 μM Y-27632/SB203580 affected TGF-β2/Smad signaling activity in HTM cells. First, we assessed the ratio of phosphorylated Smad2 to total Smad2/3, using Western blot analysis. As expected, TGF-β2 increased the ratio of phosphorylated Smad2 (95%CI: 3.493 to 9.306, P < 0.001; Fig. 4). Y-27632 tended to inhibit TGF-β2–induced Smad2 phosphorylation, although the effect was not significant. Ten μM SB203580, however, significantly inhibited TGF-β2–induced Smad2 phosphorylation (95%CI: 0.724 to 6.537, P = 0.011), but not to basal levels. Next, we observed nuclear translocation of Smad2/3 using immunocytochemistry. TGF-β2–induced nuclear translocation of Smad2/3 was not affected by pretreatment with 10 μM Y-27632, but was partially inhibited by 10 μM SB203580 (Fig. 5A-5F). This effect was further confirmed by assessment of nuclear and cytoplasmic protein extractions, and the estimation of the relative expression of nucleic Smad2 to its cytoplasmic expression (Fig. 5G). Finally, we investigated the transcriptional activity of R-Smad in HTM cells, using the CAGA12-luciferase (CAGA12-Luc) reporter gene, which includes a 12-time repeat of the Smad complex binding sequence. CAGA12-Luc reporter activity was significantly increased by TGF-β2 treatment in HTM cells. This effect was significantly inhibited by pretreatment with 10 μM SB203580 (95%CI: 0.361 to 6.773, P = 0.021), but not 10 μM Y-27632 (Fig. 6). These results indicate that SB203580 partially suppresses the TGF-β2–induced activation of R-Smad, and the subsequent induction of Smad target genes, in HTM cells.

## Discussion

Both TGF-β2 and Rho-ROCK signaling pathways are involved in regulating aqueous outflow, and are therefore important in glaucoma pathology. Since TGF-β2 increases actin stress fibers, and TGF-β2–induced fibronectin production is inhibited by the suppression of Rho-ROCK signaling [8], TGF-β2 is suggested to activate Rho-ROCK signaling in TM cells. In the present study, we show that TGF-β2 directly activates RhoA, and that a ROCK inhibitor suppresses TGF-β2–induced polymerization of actin stress fibers, whereas a p38 inhibitor has almost no effect. In agreement with the present study, another TGF-β family protein, TGF-β1, has been shown to induce collagen contraction in bovine TM cells. In these cells, thicker actin stress fibers are formed compared with control cells, and chemical inhibition of Rho-ROCK signaling suppresses gel contraction [23]. These results suggest that Rho-ROCK signaling, but not p38 MAP kinase signaling, is involved in TGF-β2–induced actin polymerization, at least in the context of HTM cells.

In human TM cells, there is reportedly a link between Rho GTPase and TGF-β2 signaling pathways [24]. In addition, collagen production without external TGF-β stimulation is reported to be suppressed by a ROCK inhibitor, fasudil, in hepatic stellate cells [25]. The present study, however, demonstrates that the corresponding effect of an another ROCK inhibitor, Y-27632, is limited in HTM cells. The causative mechanisms behind this difference are unknown. There might be context-dependent differences among cell lines or tissues. Alternative explanations are related to differences in ROCK selectivity between fasudil and Y-27632, and the influence of external TGF-β2. Further study using genetic modulations, such as the knock-down of the target genes and the induction of C3 exoenzyme, will be required to elucidate the complete role of Rho-ROCK signaling in TGF-β2–induced production of type 1 collagen in HTM cells.

TGF-β activates several MAP kinase signaling pathways, such as the p38, PI3K-Akt, ERK, and JNK pathways [26]. Among them, the biochemical blockade of p38 MAP kinase activation, but not ERK activation, inhibits TGF-β2–induced type I collagen synthesis in retinal epithelial cells [25]. Consistent with these previous findings, the present study demonstrates that SB203580, a p38 inhibitor, suppresses TGF-β2–induced synthesis of type 1 collagen more effectively than Y-27632 in HTM cells. The inhibitory effects were presented at every level: promoter, mRNA and protein. These results suggest that p38 MAP kinase signaling is involved in TGF-β2–induced synthesis of type 1 collagen in HTM cells. Similarly, it is reported that TGF-β2–induced expression of secreted protein acidic and rich in cysteine (SPARC) is suppressed by the inhibition of p38 in HTM cells [27]. In addition, Sethi *et al*. showed that gremlin, a BMP inhibitor, activates TGF-β signaling and thereby induces lysyl oxidase genes in HTM cells [28]. In their report, the induction of lysyl oxidase genes is shown to be suppressed by p38 inhibitor, SB203580.

Activation of R-Smad proteins is essential for signal transduction in the canonical TGF-β pathway. ECM production is also thought to be induced by R-Smad activation and transcription of its target genes. Inactivation of the Rho-ROCK signaling pathway suppresses the canonical TGF-β pathway in some cell types, such as retinal epithelial cells, smooth muscle cells, and mesenchymal stem cells [22], [29], [30]. In the present study, however, the inhibitory effect of Y-27632 on R-Smad activation was not observed. Interestingly, previous studies reported that TGF-β–induced Rho activation was Smad-dependent in some cells [31], [32]. If Rho-ROCK signaling in TM cells is involved in TGF-β2 signaling after activation of R-Smad proteins, this might explain why Y-27632 did not block R-Smad activation in HTM cells.

Corticosteroids are widely used for the treatment of ocular inflammatory diseases. Armaly reported that the glucocorticoid dexamethasone (DEX) induces changes in IOP and fluid dynamics more frequently in glaucomatous than in normal eyes [33], [34]. We reported previously that DEX also activates RhoA in HTM cells and that the induction of *fibronectin* and *COL4A1* mRNAs are inhibited by Y-27632 [20]. In the previous study, however, DEX did not induce *COL1A1* mRNA. Thus, both TGF-β2 and DEX induce ECM production through activation of Rho-ROCK signaling, but the downstream regulatory mechanisms are suggested to be different.

In conclusion, TGF-β2–induced production of type 1 collagen in HTM cells is suppressed by p38 inhibition and accompanied by partial inactivation of Smad2/3. The effect of ROCK inhibition on this induction is limited and not associated with significant effects on Smad2/3 activation.

## Supporting Information

S1 Fig The characters of HTM cells in the present study. (A) Immunocytochemical detection of TM cell specific proteins. Scale bar: 50 μm. (B) Phase contrast photos of HTM cells with phagocytosed particles (pHrodo bioparticles; green) and nuclear staining (Hoechst33342; blue). Scale bar: 50 μm. (C) Induction of myocilin in HTM cells by dexamethasone treatment. *P < 0.05 calculated using the t-test test.
(TIF)

## Abbreviation

DEX: dexamethasone
MGP: matrix protein gla
TIMP3: tissue inhibitor of metalloproteinase 3
Vcam1: vascular cell adhesion protein 1
