# Supplementary figures and images for: p38 MAP Kinase Inhibitor Suppresses Transforming Growth Factor-β2–Induced Type 1 Collagen Production in Trabecular Meshwork Cells
Source: PLoS One. 2015 Mar 23;10(3):e0120774. doi: 10.1371/journal.pone.0120774 (PMC4370581; doi:10.1371/journal.pone.0120774)

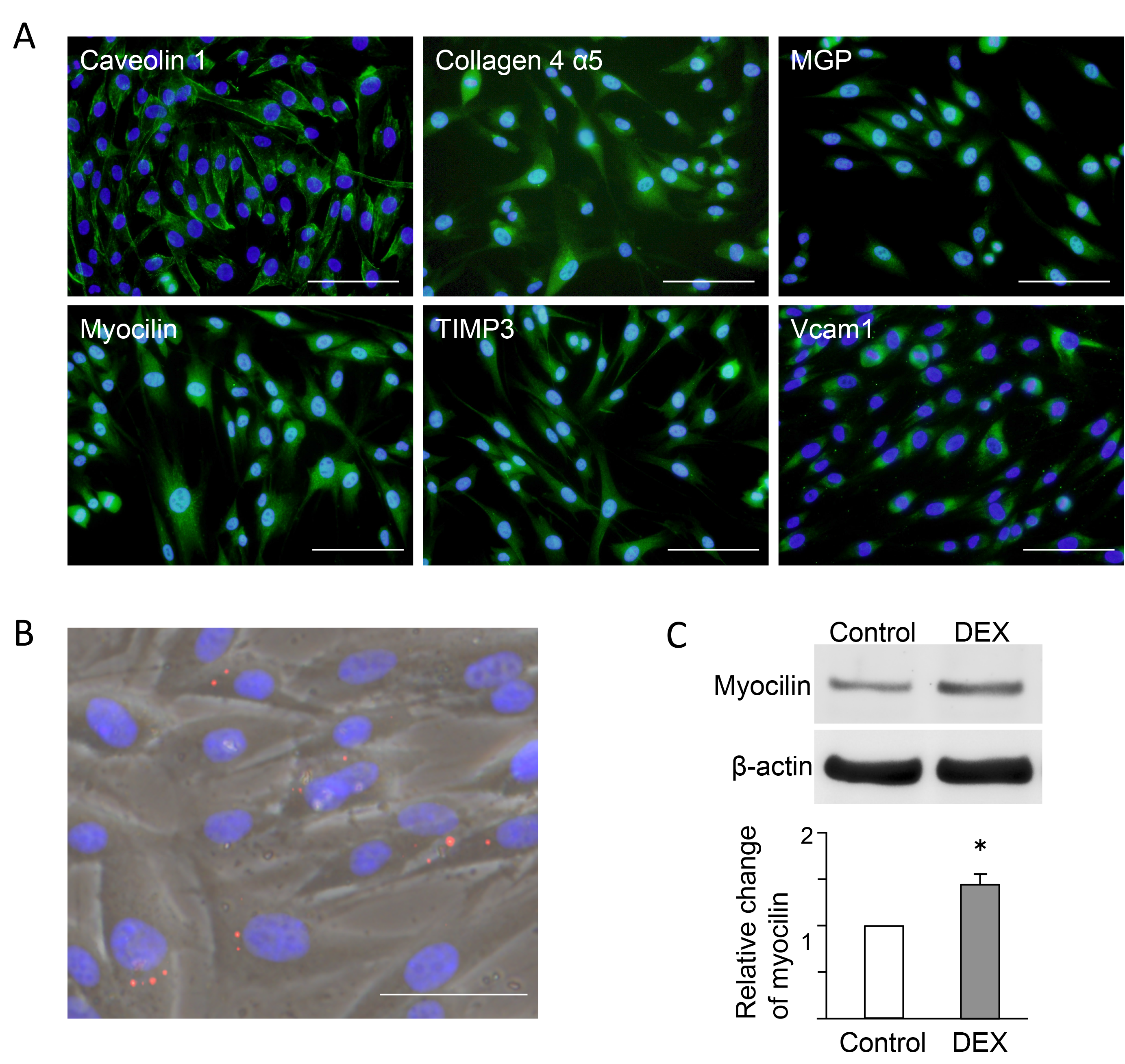

Supplement: S1 Fig — (A) Immunocytochemical detection of TM cell specific proteins. Scale bar: 50 μm. (B) Phase contrast photos of HTM cells with phagocytosed particles (pHrodo bioparticles; green) and nuclear staining (Hoechst33342; blue). Scale bar: 50 μm. (C) Induction of myocilin in HTM cells by dexamethasone treatment. *P < 0.05 calculated using the t-test test. (TIF) [file pone.0120774.s001.tif]
